# Centring women’s voices in contraceptive innovation: building the case for an on-demand, pericoital pill

**DOI:** 10.1136/bmjsrh-2024-202510

**Published:** 2024-11-26

**Authors:** GIlda Sedgh, Laura J Frye, Kristina Gemzell-Danielsson, Nathalie Kapp, Kayode Afolabi, Angela A Boateng, Mary Mulombe-Phiri, Sharon Cameron, Kanya Manoj, Kirti Iyengar, Abigail Grace Winskell, Kristen M Little, Susannah Gibbs, Eden Demise, Stephen Bell

**Affiliations:** 1 Evidence & Learning Working Group, Self-Care Trailblazers Group, Philadelphia, Pennsylvania, USA; 2 FHI 360, Durham, North Carolina, USA; 3 Women's and Children's Health, Karolinska Institute, Stockholm, Sweden; 4 International Planned Parenthood Federation, London, UK; 5 Independent Consultant, Abuja, Nigeria; 6 Ghana Health Service, Accra, Ghana; 7 Reproductive Health Directorate, Government of Malawi Ministry of Health, Lilongwe, Malawi; 8 Sexual and Reproductive Health, NHS Lothian, Edinburgh, UK; 9 University of Edinburgh Division of Health Sciences, Edinburgh, UK; 10 Children's Investment Fund Foundation, London, UK; 11 Sexual & Reproductive Health, Population Services International, Washington, District of Columbia, USA; 12 Strategy & Insights Department, Population Services International, Washington, District of Columbia, USA; 13 Independent Consultant, Melbourne, Victoria, Australia; 14 Women’s, Children’s and Adolescents’ Health, Burnet Institute, Melbourne, Victoria, Australia

**Keywords:** scoping review, on-demand contraception, family planning, contraceptive preferences, emergency contraception, pericoital contraceptive, reproductive health, new contraceptive methods, sexual agency

 Despite growing support for reproductive autonomy globally^1 2^ and the push to achieve the United Nations (UN) Sustainable Development Goals Target 3.7 (ensuring universal access to sexual and reproductive healthcare services), progress in meeting people’s contraceptive needs has been slow. Recent estimates indicate that 218 million women of reproductive age in low- and middle-income countries still have an unmet need for modern contraception – that is, they wish to avoid a pregnancy but are not using a modern method.^3^

While the level of unmet need for contraception has hardly shifted in recent years, women’s reasons for not using a contraceptive method have changed.^4^ In 2016, an analysis of survey data from 39 countries from 2000 onwards^4^ found that a small, shrinking proportion of women say they lack knowledge of contraception or access to contraceptive care, while a sizeable, growing proportion of women say they are not using a method because those available do not suit their needs. Infrequent or no sex was one of the two most cited reasons for not using a method (along with concerns about method side effects or health risks), mentioned by 24% of married women and 41% of unmarried women.^4^ Among their recommendations, the authors suggested that investments in new, more user-aligned, contraceptive methods could address women’s needs and concerns.^4^

Development of an ‘on-demand’ (defined as coitally-dependent usage) oral contraceptive pill that could be taken pericoitally (ie, before or after sex) represents one such innovation. Several recent studies have contributed to the body of evidence suggesting that these on-demand pills can be efficacious,^5 6^ feasible,^7 8^ acceptable^6^,^8^ and safe with limited side effects.^6^,^8^ Although on-demand pericoital pills may be less effective at preventing pregnancy than many available long-acting reversible contraceptives, some women and other individuals at risk of pregnancy are likely willing to make trade-offs between level of effectiveness and the ability to avoid some side effects, the costs associated with use of a continuous method, and to have control over when they start and stop using a method. For those people having infrequent sex, who are already at a lower overall risk of pregnancy, the impact of a less effective method may be more limited.

A recently published systematic scoping review of 30 papers published between 2014 and 2023 from 16 countries across five WHO regions indicates widespread appeal among women for an on-demand contraceptive pill.^9^ The analysis found that use of an on-demand pericoital contraceptive pill aligned more closely with some women’s sexual lives, particularly those who reported having occasional or spontaneous sex, or who could not plan for sex. The review also found that discreet, routine postcoital use of the emergency contraceptive pill (further supported by convenient over-the-counter availability in many settings) enabled some women to navigate circumstances in which their reproductive agency was constrained.

Together, these rationales point to the potential benefits of an on-demand contraceptive pill to satisfy the demand for contraception among many non-users at risk of unintended pregnancy, and to support their agency in their sexual and reproductive decision-making and action. Regular use of available postcoital contraceptive pills could address some of these barriers, and introduction of a pericoital product that could be used before or after sex as a person’s main form of contraception would have several advantages. These include the option for advanced provision, availability of a method less burdened by stigma around ‘over-use’ of a product intended only for emergencies, and the ability to enter potential sexual encounters knowing they have already taken measures to prevent unintended pregnancy.

Further contraceptive innovation is required to expand the range of affordable, acceptable and accessible contraceptive products and technologies that enhance women’s and girls’ control over their own contraceptive care, in ways that meet their needs. The addition of an on-demand contraceptive pill to the current method mix responds not only to longstanding global calls, commitments and movements for sexual and reproductive justice and equity, but also to the voices of women themselves.

We encourage funders to recognise the potential of on-demand contraceptive products, support ongoing work to confirm their safety, efficacy and user acceptability, and bring such a product to market. More broadly, we encourage research on new methods, and innovations that leverage existing contraceptive technologies, to meet the needs of people whose circumstances are not well-aligned with the current range of contraceptive options. Together, these efforts have the potential to fill a critical gap in contraceptive markets for those who currently find themselves dissatisfied with current contraceptive options that do not align with their lived experiences and expressed needs.
